# An evaluation of the Beckman Astra 8 analyser

**DOI:** 10.1155/S1463924680000429

**Published:** 1980

**Authors:** P. H. Lloyd, Helen Bicknell, P. M. G. Broughton

**Affiliations:** Wolfson Research Laboratories Dept of Clinical Chemistry Queen Elizabeth Medical Centre Edgbaston Birmingham B15 2TH UK


### An evaluation of the Beckman Astra 8 analyser

**P. H. Lloyd, Helen Bicknell and P. M. G. Broughton***

*Wolfson Research Laboratories, Dept of Clinical Chemistry, Queen Elizabeth Medical Centre, Edgbaston, Birmingham B15 2TH.*

### Introduction

The Astra-8 is a floor-standing seven-channel, automatic, discrete analyser for use in the clinical chemistry laboratory. At present it will analyse serum for urea, sodium, potassium, chloride, total carbon dioxide, glucose, and creatinine. It calculates the anion gap and osmolality, and also the ratio of blood urea nitrogen to creatinine when these are expressed as mg/dl. Urine may be analysed for sodium, potassium, chloride, glucose and creatinine. Provision is made for the addition of two further analytical modules when these become available.

The sample tray holds up to 38 specimens and two calibrating solutions, and requests for up to 76 specimens may be entered into the machine at one time. Any combination of available tests may be requested for each specimen or group of specimens, but sodium and potassium are always measured together. The machine may be instructed to analyse an emergency specimen at any time, after which it will return to the original sequence of specimens.

The analyser is controlled by a computer to which the operator communicates by means of a keyboard and a visual-display unit (VDU). The operator can select individual tests for each specimen, enter identification numbers, enter or alter reference ranges, initiate priming of reaction vessels and automatic calibration on all or any channels, request duplicate assays, and to return to a control specimen at any desired frequency. Special procedures for routine maintenance or diagnosis of faults can be initiated from the keyboard. In addition the computer monitors the functioning of the instrument, detects the presence of a sample in the cup, converts the outputs from the analytical modules to analytical results, expressed either as mass (eg mg/dl) or molar (eg mmol/l) concentrations. Results are displayed on the VDU and printed on a high-speed thermal printer in a form which could be used as the report. This includes the date and time of the analysis, patient identification number, the analytical results, the reference ranges, and an indication if the result is outside that range. The analyser may also be connected to external data-processing equipment via a multipin socket.

Reagents provided by the manufacturer are stored on top of the machine. They are pumped to the reaction vessels by peristaltic pumps via preheating elements, and excess is removed via an overflow. Although the principle components of each reagent are listed on the bottle, insufficient detail is given to enable the user to prepare them.

Sodium and potassium are measured by means of ion-selective electrodes (lithium aluminium silicate glass and valinomycin respectively). The reference electrode is a second sodium electrode in contact with a sodium solution of constant concentration. The electrodes are equilibrated prior to measurement, and 50μl of serum or urine are required.

Chloride is measured by coulometric titration with silver ions released from a silver anode. It requires 8μl of serum or 6μl of urine.

Glucose is estimated by measuring the rate of consumption of oxygen in the presence of glucose oxidase by means of an oxygen electrode. It requires 10μl of serum or urine.

**Address correspondence to this author*

Urea is measured from the rate of change of conductivity in the presence of urease. It requires 10μl of serum. Urine can be analysed if prediluted with saline to bring the urea concentration within the range for serum.

Total carbon dioxide is measured by the addition of sulphuric acid. The carbon dioxide released diffuses through a membrane and the rate of change of pH in a bicarbonate buffer is measured. It requires 12μl of serum.

Creatinine is measured by mixing the sample with alkaline picrate solution and measuring the rate of change of absorbance at 520 nm. It requires 30μl of serum or 10μl of urine.

The minimum volume of serum required for all seven tests is 175μl.

Two aqueous calibration standards provided by the manufacturers are each analysed at least twice, and the result of each analysis is displayed on the basis of the previous calibration. If the electrical signals satisfy predetermined criteria, a two-point calibration is performed. If these criteria are not satisfied each standard may be analysed up to four times after which an alarm sounds and the operator must intervene. In this case the reason for the failure to calibrate is displayed on the VDU, and the operator can then use diagnostic routines held within the computer to identify the cause. Forty minutes after a calibration, a warning light flashes to indicate that the machine will soon need recalibration. If no action is taken, the instrument will stop analysing ten minutes later. The operator can either initiate a new calibration or override the alarm, after which the machine will continue analyses for a further 50 minutes. If calibration has been overriden, the letters COR are printed on the report.

A smaller bench-mounted version of the system, the Astra-4, is available. This has four analytical modules and no VDU, but has not been examined here.

The RAPid kit is available as an optional extra. This consists of spare analytical modules and electronic boards so that in the event of failure the defective unit may be replaced by the user. The modules are designed to be easy and quick to replace and the chemistry modules (but not the electronic boards) may be replaced without switching the instrument off, thereby retaining volatile memory within the computer.

A comprehensive manual is provided which describes the operation and maintenance procedures. The manufacturers also arrange training courses for the instrument.

### The evaluation

The instrument was obtained on loan from the manufacturers (Beckman-RIIC Ltd, Cressex Industrial Estate, Turnpike Road, High Wycombe, Bucks, UK) and evaluated in the authors' laboratories between 11 May and 27 June 1979. Before commencing the evaluation, one of the authors attended the manufacturers' training course.

The evaluation followed a recommended protocol as described by Broughton *et al* [1], although the nature of the sampling and measurement systems made independent assessment of these impracticable. The instrument was therefore evaluated as a complete system using reagents and standards supplied by the manufacturer. The aspects evaluated included precision, linearity, accuracy, interaction between channels, and correlation with routine methods. Tests for interference were made with haemoglobin, bilirubin, methyl dopa, ethylene-diamine-tetraacetic acid (EDTA), heparin and fluoride-oxalate. In addition, the effects of hydrochloric acid and ammonia on urine assays were examined. Carryover between specimens and from wash fluid to specimens was also estimated.

At the end of the main evaluation, three chemistry modules, the sampling module and two electronic boards were replaced from the RAPid kit to assess the ease of this procedure. The within-batch precision and linearity of the system were then rechecked.

### Methods

### Precision

A normal, human quality control serum was reconstituted with either water or solutions containing additional analytes in order to obtain the required concentrations (see Results). Another control material with low values of most analytes was used for the lower concentrations. Aliquots of pools of these sera were analysed twenty times within a single batch (and within one calibration period). Aliquots were also stored at −20°C and one of each level analysed on each of twenty working days. The concentrations of each constituent spanned the analytical range and included clinically important levels.

### Linearity

Aqueous solutions were prepared containing eleven different concentrations of one analyte, spanning the analytical range for serum or urine, and constant concentrations of all other constituents. These solutions were used to test linearity and any interactions between channels. It was not possible to keep the chloride concentration constant in the presence of varying sodium or potassium concentrations or vice versa, since this would have required nonphysiological levels of other ions in order to maintain electrical neutrality.

A serum with high concentrations of all analytes was diluted volumetrically either with water or with another serum containing relatively low levels to obtain mixtures with linearly-related concentrations.

### Accuracy

Standard solutions were prepared by weighing dried AnalaR reagents and dissolving these either in water or physiological saline.

Seven lyophilised sera which had been circulated through the UK National Quality Control Scheme (NQCS), and three that had been assayed in the Wellcome Group Quality Control Programme (WGQCP), were analysed on the Astra and the results compared with the consensus values from these schemes.

### Comparison with routine methods

Approximately 500 specimens of serum submitted for routine analysis by the methods listed in Table 1 were also analysed on the Astra. Only 100 assays for chloride were done, since in the authors' laboratory chloride analyses are not performed in large numbers. No comparison for total carbon dioxide was made.

### Carryover

Sample-to-sample carryover was measured using sera with high and low concentrations of all analytes [1].

Since the sample probes are washed before and after every sampling, carryover from wash fluid to samples was assessed by adding procion blue to the wash fluid and measuring any increase in absorbance of the samples.

### Interferences

The effects of anticoagulants were assessed by dividing a blood specimen from a normal volunteer into four aliquots. One was allowed to clot to produce serum, and an anti-coagulant was added to each of the other samples and the plasma separated. The four specimens were then analysed on all channels.

**Table 1. Methods used for comparison**

| Analysis | Instrument | Method |
|---|---|---|
| Urea | SMA 12/60 | Diacetylmonoxime |
| Sodium | IL 543 | Flame photometry |
| Potassium | IL 543 | Flame photometry |
| Chloride | EEL 920 | Coulometric titration |
| Glucose | SMA 12/60 | Glucose oxidase |
| Creatinine | SMA 12/60 | Jaffé end point |
| Creatinine (urine) | Autoanalyser | Jaffé end point |

**Table 2. Precision results for serum**

| Analyte | | Within-batch | | | | Between-batch | | | |
|---|---|---|---|---|---|---|---|---|---|
| | | Low | Med | High | V High | Low | Med | High | V High |
| Urea | Mean | 2.59 | 7.25 | 17.88 | 38.17 | 2.71 | 7.22 | 18.15 | 38.48 |
| (mmol/l) | SD | 0.16 | 0.17 | 0.16 | 0.20 | 0.21 | 0.34* | 0.36 | 1.46 |
| | CV% | 6.31 | 2.30 | 0.90 | 0.53 | 7.72 | 4.70* | 1.99 | 3.79 |
| Sodium | Mean | 124.05 | 144.90 | 164.20 | 201.75 | 121.95 | 143.40 | 163.80 | 201.80 |
| (mmol/l) | SD | 0.83 | 0.55 | 0.41 | 0.55 | 0.95 | 0.88 | 1.47 | 1.51 |
| | CV% | 0.67 | 0.38 | 0.25 | 0.27 | 0.77 | 0.62 | 0.90 | 0.75 |
| Potassium | Mean | 2.70 | 4.00 | 5.99 | | 2.69 | 4.10 | 6.10 | |
| (mmol/l) | SD | 0.02 | <0.02 | 0.02 | | 0.04 | 0.06 | 0.06 | |
| | CV% | 0.83 | <0.5 | 0.37 | | 1.36 | 1.58 | 0.99 | |
| Chloride | Mean | 82.05 | 105.05 | 125.30 | 141.3 | 83.25 | 105.70 | 126.40 | 141.80 |
| (mmol/l) | SD | 0.51 | 0.51 | 0.73 | 0.73 | 1.12 | 2.25 | 2.70 | 2.28 |
| | CV% | 0.62 | 0.49 | 0.58 | 0.52 | 1.34 | 2.13 | 2.14 | 1.61 |
| Carbon dioxide | Mean | 11.41 | 15.59 | 29.33 | | 10.51 | 14.31 | 27.94 | |
| (mmol/l) | SD | 0.14 | 0.10 | 0.39 | | 0.33 | 0.51 | 0.94** | |
| | CV% | 1.27 | 0.65 | 1.34 | | 3.14 | 3.57 | 3.37** | |
| Glucose | Mean | 1.91 | 4.26 | 7.53 | 19.13 | 1.87 | 4.26 | 7.60 | 18.92 |
| (mmol/l) | SD | 0.04 | <0.05 | 0.09 | 0.18 | 0.06 | 0.08 | 0.09 | 0.29 |
| | CV% | 1.91 | <1.18 | 1.13 | 0.93 | 3.41 | 1.77 | 1.17 | 1.55 |
| Creatinine | Mean | 89.15 | 154.70 | 300.10 | 1848.50 | 95.70 | 149.40 | 291.25 | 1806 |
| (µmol/l) | SD | 3.47 | 4.41 | 2.79 | 6.22 | 5.20 | 2.87 | 5.79 | 24.4 |
| | CV% | 3.89 | 2.85 | 0.93 | 0.34 | 6.07 | 1.92 | 1.99 | 1.35 |

**These results include a result 6.8 SDs below the mean of the other 19 results. When this result is omitted, the SD is 0.19 and the CV is 2.6%.*
***Results showed a downward drift, possibly caused by instability of the specimen.*

The effects of haemolysed erythrocytes, bilirubin, and methyl dopa were assessed by adding each to a serum pool.

The effects of added hydrochloric acid and ammonia on urine assays was also assessed.

### Serum osmolality
This was calculated by the Astra and compared with values measured by an Osmette osmometer (Precision Systems, Shuco Scientific Ltd, Halliwick Court Place, Woodhouse Road, London N12, UK).

### Results
### Precision
The results of the precision studies are given in Tables 2 and 3. In a few instances the resolution of the printed result was such that a reliable estimate of the standard deviation (SD) could not be obtained. In these cases an upper limit for the true SD was estimated and this is given in the tables together with the less-than sign.

### Linearity
Using aqueous solutions, results for all channels except total carbon dioxide were found to be linear over the ranges stated by the manufacturer. Although the deviation from linearity for carbon dioxide was statistically significant it represented a maximum error of only about 1 mmol/l which would not be significant clinically.

When serum was diluted with serum, non-linearity was found for urea above approximately 30 mmol/l amounting to about 5 mmol/l low at 50 mmol/l, and for carbon dioxide above about 35 mmol/l amounting to approximately 3 mmol/l low at 50 mmol/l.

### Accuracy
Recoveries from standard solutions were between 98.5 and 101.5%. The results of the comparison with external quality control schemes are given in Table 4.

### Comparison with routine methods
Graphs showing the comparison between the Astra results and those obtained by routine methods are shown for creatinine in serum in Figure 1, and for potassium in urine in Figure 2. Essentially similar graphs were obtained for the other constituents. The coefficients of the regression equations given in Table 5 include the ratios of variances used in their computation [2].

### Carryover
Carryover from one specimen to the next was undetectable even when serum followed urine. Carryover from wash fluid to samples was less than 1 μl.

### Interferences
Apart from the expected interferences of fluoride-oxalate and EDTA on sodium and potassium measurements, the only interferences observed from anticoagulants were that fluoride-oxalate produced an 11.8% reduction in creatinine at a level of 87μmol/l; lithium heparin caused a 5.6% reduction in potassium at a level of 4.3 mmol/l; EDTA produced a reduction of 10.7% in carbon dioxide at 29 mmol/l and a 6.3% reduction in chloride at 105 mmol/l.

Of the other potentially-interfering substances studied, bilirubin caused a small increase in total carbon dioxide concentration (5% at 534 μmol/l). It had no observable effect on creatinine up to a bilirubin concentration of 430 μmol/1, but produced an apparent decrease of 20% at a level of 680μmol/l.

Haemolysis at 10 g/l haemoglobin produced a small decrease in urea and creatinine which was not statistically significant.

Methyl dopa caused a 7% increase in creatinine when present at 12.5 or 25 mg/l and a 20% increase at 75 mg/l. The latter level of methyl dopa is ten times that recorded following intra-venous infusion; normal therapeutic levels are usually less than 2.6 mg/l.

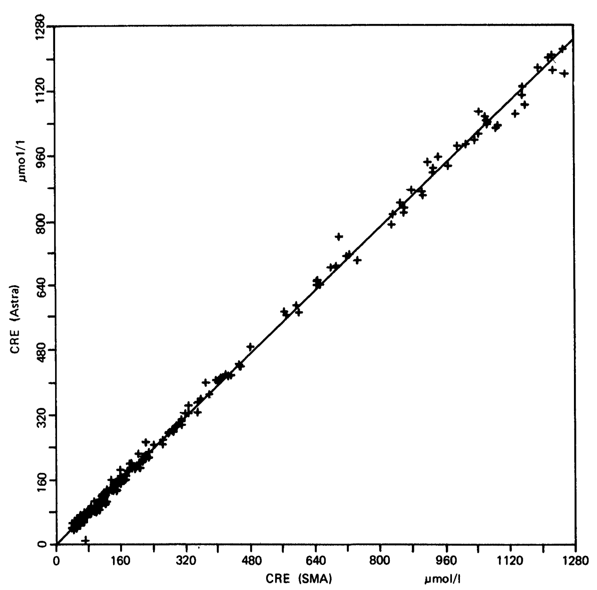

*Figure 1. Comparison of creatinine in serum*

**Table 3. Precision results for urine**

| Analyte | | Within-batch | | | Between-batch | | |
|---|---|---|---|---|---|---|---|
| | | Low (mmol/l) | Med (mmol/l) | High (mmol/l) | Low (mmol/l) | Med (mmol/l) | High (mmol/l) |
| Sodium | Mean | 19.5 | 152.20 | 219.90 | 18.35 | 152.50 | 215.55 |
| | SD | <0.5 | 0.89 | 0.85 | 1.04 | 1.70 | 2.93 |
| | CV% | <2.6 | 0.59 | 0.39 | 5.67 | 1.12 | 1.36 |
| Potassium | Mean | 15.23 | 49.14 | 142.95 | 15.42 | 51.07 | 141.30 |
| | SD | 0.11 | 0.40 | 1.05 | 0.25 | 1.00 | 4.23 |
| | CV% | 0.71 | 0.82 | 0.73 | 1.59 | 1.96 | 2.99 |
| Chloride | Mean | 16.70 | 140.35 | 299.80 | 14.88 | 142.10 | 294.05 |
| | SD | 0.73 | 0.99 | 1.28 | 2.18 | 2.97 | 4.11 |
| | CV% | 4.39 | 0.70 | 0.43 | 14.62 | 2.09 | 1.40 |
| Glucose | Mean | 4.64 | 11.64 | 22.09 | 4.60 | 11.70 | 21.41 |
| | SD | <0.05 | 0.17 | 0.27 | 0.11 | 0.21 | 0.37 |
| | CV% | <1.08 | 1.46 | 1.21 | 2.34 | 1.82 | 1.71 |
| Creatinine | Mean | 2.49 | 11.57 | 47.70 | 2.51 | 11.65 | 46.84 |
| | SD | 0.04 | 0.06 | 0.19 | 0.08 | 0.17 | 1.02 |
| | CV% | 1.47 | 0.49 | 0.40 | 3.14 | 1.46 | 2.18 |

The linearity experiments were designed to detect any apparent interaction or interference between channels. The only effect revealed was on the measured value of urea by sodium, chloride, and bicarbonate. The effect was small but statistically significant at the 1% level of confidence. For sodium and chloride there was a decrease of 0.005 mmol/l of urea for every mmol/l of sodium or chloride. Over a range of sodium concentrations of 120-160 mmol/l the decrease in serum urea would be 0.22 mmol/l. When chloride and bicarbonate concentrations were varied together, keeping the sodium level constant, no effect on the urea result was observed. It seems likely that the cause was the variation in the ionic strength or initial conductivity of the sample rather than the sodium concentration itself, but this was not investigated further.

### Serum osmolalities

Calculated serum osmolalities are compared with measured values in Figure 3. There is broad agreement between the calculated and the measured values but with one or two grossly outlying points, especially for a haemolysed sample.

### Outlying results

During the evaluation approximately 2000 analyses were performed on each channel. Some gross outlying results were obtained for which there was no obvious cause. During the between-batch precision studies, a result for serum urea of 6.0 mmol/l was obtained, which was 6.8 SD's below the mean of 7.3 mmol/l for the other 19 results. In addition, several outliers were observed during the analysis of specimens from external quality-control schemes (Table 4):

(i) A result of 0.3 mmol/l for urea was obtained when the other four results ranged from 7.8 to 8.3, and a result of 10.7 mmol/l was obtained in a sequence ranging from 9.1 to 9.4.

(ii) Three high chloride results were obtained, but when repeated agreed with the consensus value. The machine failed to produce any result for the next five specimens, because it failed to complete the analysis in the time available, suggesting that there may have been a fault, although normal maintenance procedures had been followed. One other assay gave a chloride result of 104 mmol/l compared with 96, 96, 97, 97 for the other four.

(iii) During the carryover experiment one triplicate set of urea results were 3.0, 1.6, 3.0 mmol/l.

(iv) During the linearity study on total carbon dioxide in which the creatinine concentration was kept constant, a single result of 47µmol/l was obtained when the mean of the other 32 results was 144.4µmol/l, with a coefficient of variation of 15%. Since all the other analytes assayed precisely in this experiment, the variability of the creatinine results could not be attributed to any errors in the preparation of the solutions.

(v) If urea was measured after a period of inactivity for that channel, but within the 50-minute calibration period (such as after assaying a number of urines), low results were often obtained. It is understood that the manufacturers have taken steps to cure this problem.

### Malfunctions

During the main part of the evaluation there were no mechanical malfunctions, but there was one malfunction of the computer, when it behaved irrationally and failed to

**Table 4. Accuracy as judged by comparison with consensus values from external quality control schemes**

| Analyte | Concentration range of sera | Mean difference | Paired t | Significance* |
|---|---|---|---|---|
| Urea (mmol/l) | 3.8 – 28.2 | – 0.44 | 2.74 | S |
| Sodium (mmol/l) | 128 – 161 | + 2.88 | 5.41 | VS† |
| Potassium (mmol/l) | 3.0 – 8.1 | + 0.04 | 1.27 | NS |
| Chloride (mmol/l) | 89 – 120 | + 1.70 | 4.87 | VS |
| Carbon dioxide (mmol/l) | 17.5 – 23 | – 0.71 | 2.63 | S |
| Glucose (mmol/l) | 3.0 – 17.4 | + 0.002 | 0.03 | NS |
| Creatinine (µmol/l) | 71 – 827 | – 6.0 | 2.26 | NS |

Five replicate analyses were made on 10 sera (3 for carbon dioxide) and mean values for each were compared with the quoted consensus values.

* *NS = Not significant at 5% level*
*S = Significant at 5% level*
*VS = Significant at 0.1% level*

† *These results may have been affected by the problems encountered with this channel (see Discussion).*

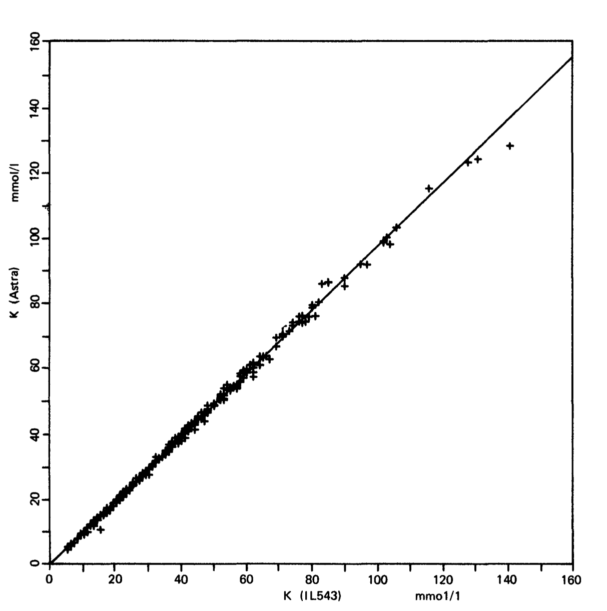

*Figure 2. Comparison of potassium in urine*

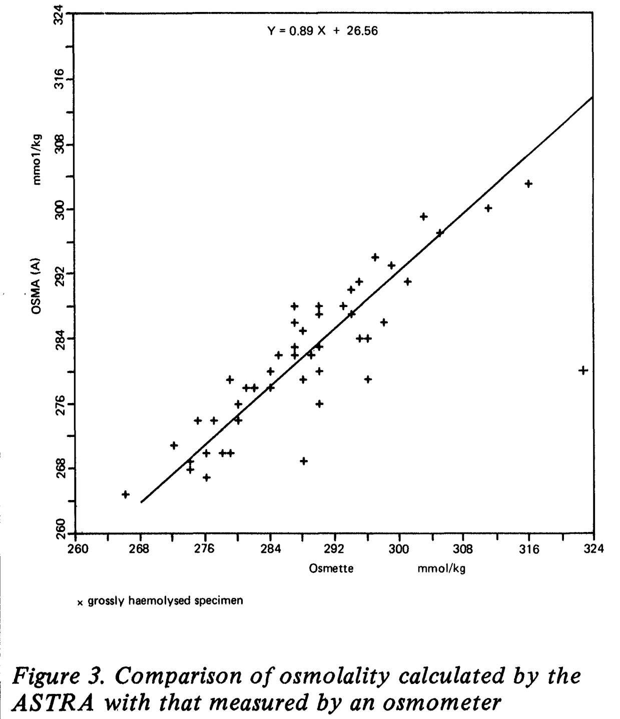

*Figure 3. Comparison of osmolality calculated by the ASTRA with that measured by an osmometer*

respond to commands from the keyboard. The cause was never established. Pressing the general-reset button cured the problem but caused all the volatile memory within the computer to be lost.

After the main evaluation was finished, there occurred two malfunctions of the transport system which carried the samples to the analytical modules. In both cases the problem was cleared from the keyboard and the instrument continued to operate satisfactorily. However, the machine did not re-assay the specimen on which it was working at the time and so these results would have been lost if the operator had not specifically instructed the machine to return to this specimen as though it was a new one.

A further intermittent fault occurred in the transport system on three occasions. Each time the fault cured itself before the cause could be established.

### The RAPid kit

Four modules and two electronic boards from the RAPid kit were replaced to assess the ease of doing this. The procedure was simple and took about 30 minutes for a module, most of which time was spent flushing reagent out of the "defective" module and repriming the new one. Replacing the electronic boards was also easy, taking about 5 minutes. This involved turning the machine off, so losing volatile memory within the computer.

### Electrical and mechanical construction

Members of the engineering staff examined the machine without making rigorous mechanical and electrical tests.

The electronic construction and the components used were judged to be well up to normal commercial practice. An interlock prevented removal of a perspex cover over the electronics assembly without switching off the power. Fuses were labelled with the correct rating and a notice drew attention to the importance of replacing fuses with the correct type.

There were adequate precautions to ensure that reagents spilled in the storage area or leaked from any chemistry modules would run to waste without entering the electronics assembly. However, the working area was not well protected against spillage of samples penetrating to places where it would be difficult to remove. The actual surface was of a type that would wipe clean, but spillages might, for example, run under the cover of the keyboard. Although trays may be loaded remote from the machine, the need to key in requests and patient identification numbers at the keyboard would make it tempting to load the tray whilst it was in position on the instrument.

When the main cover is in place over the analytical modules, the only moving parts to which the operator has access are the sample tray and the sampling probes. However, neither of these represented any hazard to the operator.

### Cost of reagents

The cost of reagents depends very much on the workload because reagent is consumed during calibration and automatic-priming cycles. During the night or weekend, if the machine is not required, most (but not all) reagents can be replaced by water. Ignoring consumption of reagents for these purposes, the cost per test varies between 2.4p (chloride) and 6p (urea). In this evaluation the costs were approximately twice these figures since the machine was not subjected to a big workload and comparatively large volumes were used for priming and calibration.

### The training course

A training course for two operators is included in the purchase price of the instrument. The course provided a comprehensive guide to both the operation and maintenance of the Astra system. A booklet provided before the course explained the basic features of the instrument. The course itself included detailed instructions in the principles of each analytical method, the operating procedures and trouble shooting. The operator was given a good theoretical and practical training in the use of the system and was able to use the instrument with confidence after its installation.

### Discussion

The precision of the instrument on some channels was better than the resolution of the printer, so that some of the SDs found were either zero or larger than the correct values. In either case it has been possible using simulated results to estimate an upper limit above which the true SDs probably do not lie, and these are indicated in the tables by the 'less-than' sign.

### Sodium

This was the only channel with which real difficulties were experienced. Although potassium is measured within the same module and uses the same reference electrode, no such problems were experienced with this test.

During runs of 40 specimens of different sera, there was evidence of drift but this did not seem to occur when analysing aqueous solutions or the same serum repeatedly. The manufacturer was informed, and the module was cleaned by an engineer who also improved certain electrical connections and replaced both electrodes. It is not possible to be certain that these modifications removed this problem, but it has not been mentioned in previous evaluation reports [3, 4, 5, 6 and 7].

### Urea

There seemed to be a disproportionate number of outliers among serum urea results, although the number is small compared with the total number of assays performed. There was little doubt that an assay performed after the module

**Table 5. The coefficients of the correlation equations relating the Astra results (y) to those of the routine methods (x)**

| | Analyte | Ratio of variances | Slope | 95% conf limits ± | Intercept mmol/l | N† |
|---|---|---|---|---|---|---|
| Serum | Urea | 7.12 | 0.96 | 0.01 | – 0.19 | 459 |
| | Sodium | 1.67 | 1.03 | 0.05 | – 2.1 | 199 |
| | Potassium | 4.74 | 1.04 | 0.01 | – 0.11 | 459 |
| | Chloride | 1.0* | 0.96 | 0.04 | 5.8 | 100 |
| | Glucose | 0.58 | 0.93 | 0.01 | 0.21 | 459 |
| | Creatinine | 1.10 | 0.97 | 0.01 | 4.6 μmol/l | 458 |
| Urine | Sodium | 1.67 | 1.00 | 0.01 | – 1.5 | 227 |
| | Potassium | 4.74 | 0.97 | 0.01 | 0.47 | 249 |
| | Creatinine | 0.80 | 1.10 | 0.01 | – 0.40 | 249 |

**Because the variance of the routine method was not known exactly, this value is assumed.*
*†Number of specimens assayed by both methods.*

had been unused for some time within the calibration period could give erroneously low results. This was most likely to happen if a series of urine samples were analysed followed by a serum, but the effect was by no means reproducible. The manufacturer is aware of this problem, which is probably caused by deterioration of the reagent in the measuring cup, and is taking steps to correct it.

### Creatinine

The Astra system seems to have overcome many of the problems associated with the kinetic Jaffe procedure. The method is precise and free from interference by bilirubin and haemoglobin except at very high concentrations. The interference by methyl dopa has been previously described [3].

### Summary and conclusions

With these reservations, the Astra was found to be a precise and accurate instrument for the measurement of electrolytes, urea, glucose and creatinine in serum and urine. It is easy to use, requiring only simple routine maintenance on a daily, weekly, and monthly routine. In the event of a failure of almost any part of the system, the RAPid kit enables the user to replace defective modules easily and quickly. It could comfortably analyse 200-300 specimens per day and is therefore particularly suitable for the medium-sized laboratory. It would also be suitable for use in larger laboratories to handle emergency specimens by day or night and to back up a larger multichannel analyser. It is similar in speed and capability to other instruments but has the advantage of analysing serum urea, creatinine, glucose, and electrolytes together and it will also analyse urine specimens.

ACKNOWLEDGEMENTS

The authors are indebted to Beckman RIIC Ltd for making the Astra-8 available for this evaluation. The assistance of Mr G Greaves and Mr R Bunce, who inspected the electrical and mechanical aspects of the machine is acknowledged, and also that of Mrs M Peters, Mr R Holder and Mr J Parekh for their help with the analysis of the results. The financial assistance of the Department of Health and Social Security is also acknowledged.

REFERENCES

[1] Broughton, P.M.G., Gowenlock, A.H., McCormack, J.J. and Neill, D.W., *Annals of Clinical Biochemistry,* 1974, **11**, 207-218.

[2] Lloyd, P.H., Annals of Clinical Biochemistry, 1978, **15**, 136-145.

[3] Finley, P.R., Williams, R.J., Lichti, D.A. and Thies, A.C., Clinical Chemistry 1978, **24**, 2125-2131.

[4] Truchaud, A., Hersant, J., Glikmanas, G. and Fievet, P., Abstracts of Papers and Posters 3rd European Congress of Clinical Chemistry, 1979, Brighton, p 44.

[5] North, J.W., Chittenden, C.G. and Macdonald, S.A. Abstracts of Papers and Posters 3rd European Congress of Clinical Chemistry, 1979, Brighton, p 44.

[6] Fievet, P. and Truchaud, A., Abstracts of Papers and Posters 3rd European Congress of Clinical Chemistry, 1979, Brighton, p 44.

[7] Finley, P.R. and Williams, R.J. Abstracts of Papers and Posters 3rd European Congress of Clinical Chemistry, 1979, Brighton, p 44.

### Notes for Contributors

### Presentation of manuscripts

Manuscripts should be typed (double-spaced) on one side of the paper only and with generous margins. The title should be brief and informative avoiding the word "new" and its synonyms. The full list of authors with their affiliations and full address(es) should appear on the title page. On a separate sheet an abstract of no more than 150 words is required. This should succinctly describe the scope of the contribution and highlight significant findings or innovations. It should be written in a style which can easily be translated into French and German.

*The Concise Oxford Dictionary* and Fowler's *Modern English Usage* (both published by Oxford University Press) should be used as the standard for spelling and grammar. Abbreviations should be limited to those generally recognised, or where a frequently occuring term is abbreviated it should, in the first instance, be explained thus "flow injection analysis (FIA) ..." and the abbreviation used thereafter. Abbreviations, for standard measures and units should follow SI recommendations. There are various publications giving guidance on the use of SI units.

References should be indicated in the text by numerals following the author's name, i.e. Skeggs [6]. On a separate sheet of paper, list all references in numerical order thus:

[6] Skeggs, L.T., *American Journal of Clinical Pathology,* 1959, **28**, 311.

Note that journal titles are given in full. Where there is more than one author, the form Foreman et al. should be used in the text but all authors should be named in the list of references. When reference is made to a chapter in a book the reference should take the following form:

[7] Malmstadt, H.V. in "Topics in Automatic Chemistry" Ed. Stockwell P.B. and Foreman J.K. 1978 Horwood, Chichester, pp. 68-70.

Only work which has been published or has been accepted for publication should be cited. Avoid the citation of documents which are subject to restricted circulation, patent literature, unpublished work and personal communications. The latter can be mentioned in the text in parenthesis.

To illustrate a paper line diagrams are preferred to photographs. Photographs should only be used when they significantly add to the discussion. Diagrams, charts and graphs should be carefully drawn in black ink on stout card or heavy quality tracing paper. Most illustrations are reduced for publication; to allow for this originals should be between 16 and 36 cm wide (the depth must not exceed 50 cm). The lettering of diagrams should be sufficiently clear to withstand reduction. Except in the case of proper names, all lettering should be in lower case print. If photographs are used they must be supplied in the form of clear, unmounted, glossy, black and white prints. "Instant" photographs are not normally acceptable. All illustrations must be identified on the reverse showing the figure number and the author's name.

Each illustration should have a fully explanitory caption. Captions should be typed together on a separate sheet of paper; they must *not* be an inseparable part of the illustration.

